# No correlative evidence of costs of infection or immunity on leucocyte telomere length in a wild population of Soay sheep

**DOI:** 10.1098/rspb.2023.2946

**Published:** 2024-04-03

**Authors:** Sanjana Ravindran, Sarah L. Underwood, Jennifer Dorrens, Luise A. Seeker, Kathryn Watt, Rachael V. Wilbourn, Alexandra M. Sparks, Rona Sinclair, Zhulin Chen, Jill G. Pilkington, Tom N. McNeilly, Lea Harrington, Josephine M. Pemberton, Daniel H. Nussey, Hannah Froy

**Affiliations:** ^1^ Institute of Ecology and Evolution, School of Biological Sciences, University of Edinburgh, Edinburgh EH9 3FL, UK; ^2^ Moredun Research Institute, Pentlands Science Park, Bush Loan, Penicuik EH26 0PZ, UK; ^3^ School of Biosciences, University of Sheffield, Sheffield S10 2TN, UK; ^4^ Institute for Research in Immunology and Cancer, Université de Montréal, Montréal, Canada H3C 3J7

**Keywords:** qPCR, St Kilda, *Ovis aries*, parasites, antibody immune response, strongyle nematodes

## Abstract

Telomere length (TL) is a biomarker hypothesized to capture evolutionarily and ecologically important physiological costs of reproduction, infection and immunity. Few studies have estimated the relationships among infection status, immunity, TL and fitness in natural systems. The hypothesis that short telomeres predict reduced survival because they reflect costly consequences of infection and immune investment remains largely untested. Using longitudinal data from a free-living Soay sheep population, we tested whether leucocyte TL was predicted by infection with nematode parasites and antibody levels against those parasites. Helminth parasite burdens were positively associated with leucocyte TL in both lambs and adults, which is not consistent with TL reflecting infection costs. We found no association between TL and helminth-specific IgG levels in either young or old individuals which suggests TL does not reflect costs of an activated immune response or immunosenescence. Furthermore, we found no support for TL acting as a mediator of trade-offs between infection, immunity and subsequent survival in the wild. Our results suggest that while variation in TL could reflect short-term variation in resource investment or environmental conditions, it does not capture costs of infection and immunity, nor does it behave like a marker of an individual's helminth-specific antibody immune response.

## 1. Introduction

Telomeres are repetitive, non-coding DNA sequences found at eukaryotic chromosomal ends that act to maintain genomic integrity and shorten with cellular replication, senescence and oxidative stress [1,2]. Telomere length (TL) of individual organisms, typically measured using a sample of whole blood cells, has emerged as an important marker of overall physiological state and past exposure to stressors [3,4]. Previous studies in both laboratory and wild animal populations suggest TL may vary with infection status and previous exposure to pathogens and could offer important insights into the physiological costs of infection, investment in immunity and immunosenescence in later adulthood [5–10]. These studies have shown how experimentally induced or naturally occurring variation in disease status and immunity are associated with TL. However, our understanding of the association between TL, infection, immunity and fitness in natural conditions remains poor. Investigating the potential role of TL in integrating information about multiple life-history traits will help us understand the mechanisms underlying variation in immunity and survival in the wild [9,11]. Here, we used a longitudinal dataset of individual TL, parasite burdens, immune phenotype and annual survival collected across two decades in a wild Soay sheep (*Ovis aries*) population to test whether infection and immune status predict TL and whether variation in TL mediates relationships between infection, immunity and survival under natural settings.

In a recent review, Giraudeau *et al*. proposed two hypotheses by which infection, immunity, ageing and TL dynamics could be related [9]. The ‘ageing cost of infection’ hypothesis suggests there could be inflammation-related processes activated in response to an infection that could directly impact TL, leading to TL attrition and faster ageing rates. Since short TLs have been shown to be associated with higher subsequent mortality risk in humans and non-model vertebrates, this could have negative consequences for future survival [12,13]. Additionally, leucocyte TL could shorten as a result of biological processes like oxidative stress or cellular ageing and lead to impaired immune function at older ages (called the ‘immunosenescence’ pathway) [9]. Immunosenescence, as a consequence of leucocyte TL shortening, can have detrimental fitness consequences increasing morbidity and mortality risk in later life [14]. The potential associations between infection, immunity, TL and survival are complex and a test of these hypotheses is vital to our understanding of whether TL dynamics, infection burdens, immune function and fitness outcomes are mechanistically linked.

Trade-offs between immunity and other life-history traits such as growth, reproduction and survival can potentially arise due to competition over a common resource pool [15,16]. TL and/or TL attrition may act as a proximate molecular mechanism mediating trade-offs between these traits and could explain how costs of infection and immunity translate into fitness costs [9,17]. However, the associations between infection burdens or immunity and TL may not reflect causal relationships: TL could covary with infection, immunity and fitness in a non-causal manner [18,19]. Previous studies in natural populations provide some evidence for increased telomere attrition rates or shorter TL in infected compared to uninfected individuals [5,6,20,21]. However, some wild studies have also found no association between parasitism and TL [22–25]. In humans, chronically infected individuals have been shown to have shorter immune cell TLs, which is further supported by evidence from an experimental study in a laboratory rodent species [10,26–28]. Studies in humans also found short TL to be associated with infectious disease mortality [13,29]. Furthermore, in an experimental study in wild birds, manipulated infection status was linked with lifespan via TL [5]. This provides support for the role of TL in mediating fitness costs of infection on life-history traits. Understanding how natural variation in parasite burdens impacts TL dynamics could shed light on the molecular processes through which infection status impacts life-history traits and their evolution.

Ageing-related processes could also contribute to shortening of leucocyte TL and lead to immunosenescence, reflecting the role of telomeres in contributing to declines in immune function with age [9,30]. For instance, shortened leucocyte TL due to early-life adversity, reproductive effort or challenging environmental conditions could contribute to dysfunctional immune responses and thereby induce an overall immunosenescent phenotype [31–35]. A previous study in elderly human adults found B- and T-cell immune responses following influenza vaccine administration to be positively associated with leucocyte TL, which suggests TL attrition may be a contributing factor to immunosenescence [14]. Additionally, immune activation due to chronic infection could result in increased leucocyte proliferation [36]. This could potentially manifest as shortened leucocyte TL since telomeres get progressively shorter with every cell division in proliferating cells [37]. TL could then act as a non-causal marker of immunological activation. Relatively few studies have characterized the relationship between TL and immune responses in the wild. A recent study in a wild bird population found TL to be positively associated with complement activity (associated with the innate immune response) across all ages [8]. However, there was no association found between TL and other innate immune response indices that are associated with recognition and elimination of pathogens [8]. Another study in a wild mammal population found no relationship between a cytokine molecule (IFN*γ*, involved in cell-mediated immunity) and leucocyte TL [38]. More evidence from natural populations is needed to establish whether TL serves as a likely molecular marker reflecting overall immune status and immunosenescence patterns. This will improve our understanding of whether TL acts as a reliable mediator of life-history trade-offs between immune-related traits and subsequent survival under natural conditions.

The Soay sheep of St Kilda are chronically infected with a number of gastrointestinal nematode parasites, with the strongyles *Teladorsagia circumcincta* and *Trichostrongylus* spp*.* being the most prevalent and damaging to their hosts [39,40]. Prevalence of helminth parasite infection in the Soay sheep is high, being nearly 100% in lambs and declining with age thereafter [39–41]. Previous studies have shown parasite burdens to predict overwinter survival and summer body weight in adult Soay sheep [39,40,42]. Mounting an antibody-mediated immune response to nematode worms plays a key role in the development of immunity in this population [43]. Previous work in this system identified *T. circumcincta*-specific plasma IgG antibody (IgG-Tc) levels to represent a pan-nematode specific antibody response [44]. IgG-Tc levels were also found to be highly repeatable, and associated with lower parasite burdens and higher probability of subsequent overwinter survival in adults, independent of other humoral and cellular immunity measures [43,45–47]. Immunosenescence has also been reported with age-related declines in *T. circumcincta*-specific IgG levels associated with decreased survival probabilities in adults ≥3 years of age [44]. Previous work also showed that leucocyte TL declines with age, is repeatable and is positively associated with survival [48,49]. However, the relationships between TL and strongyle infection, and TL and helminth-specific immunity, have yet to be explored.

This study uses existing datasets on TL, helminth parasite burdens and helminth-specific antibody immune response in the Soay sheep to understand the relationships between these physiological traits under natural conditions. We predicted leucocyte TL to be negatively associated with both parasite burdens (measured as faecal egg counts, FECS) and immune investment (IgG-Tc levels) in Soay lambs (approx. four months of age). This is because parasite exposure and immune investment are likely to result in greater fitness costs in early life than in later adulthood, due to the competing resource demands of growth and development and the immaturity of the immune response [50–52]. If such fitness costs of infection and immunity are acting via TL, we would expect a negative association between TL and infection burdens, and TL and immune response. TL could behave as a marker of an individual's helminth-specific antibody immune response since infection with helminth parasites could mean greater exposure to damage via activation of an inflammatory response or oxidative stress pathways [53–56]. In addition, mounting a strong immune response to helminth infection may require increased proliferation of B and T cells [57]. These processes together could impact TL. Since costs of parasitism have been previously reported in Soay sheep adults with immunosenescence observed in adults aged ≥3 years specifically, we predicted that, leucocyte TL will be negatively associated with parasite burdens but positively associated with IgG levels in adults ≥3 years old. Furthermore, we tested for sex-dependent costs of parasitism and immunity in lambs and adults since previous studies in this population found parasite burdens and IgG-Tc levels to vary in a sex-dependent manner [43,58]. Finally, to understand how these multiple physiological traits (parasite burdens, antibody response and leucocyte TL) are related to annual fitness, we used multivariate mixed models to estimate the covariance between these physiological traits and annual survival [44,59]. This approach allowed us to estimate the role of individual heterogeneity, annual effects and within-individual processes in the relationships between leucocyte TL, parasite burdens, immunity and survival [44,48].

## 2. Material and methods

### (a) Study system and data collection

Individual-based monitoring of Soay sheep resident in the Village Bay area of Hirta on the St Kilda archipelago has been ongoing since 1985 [60]. In April every year, around 95% of the lambs born in the study area have been uniquely tagged for identification within a few days of their birth [61,62]. Every August, temporary corral traps are built to catch as many sheep as possible (usually 50–60%) residing in the Village Bay area. This is when blood and faecal samples are collected along with measurements of morphometric traits. We used faecal and blood samples collected between 1998 and 2016 to measure parasite burdens and leucocyte TL. Data for antibody measures were from samples collected between 1998 and 2015. Mortality occurs predominantly (greater than 85%) over winter months (January–April) and regular censusing along with mortality searching allows accurate estimation of mortality dates thus providing individual-level information on age-specific survival.

### (b) Measurement of strongyle parasite burdens, antibody immune response and leucocyte TL

We included faecal samples collected within ±1 week of blood sample collection in our analyses. Faecal samples collected on same day as blood sample collection were taken directly from the rectum while those collected on a different day were obtained off pasture by following known individuals (collection usually within 1 min of defecation). Measures of leucocyte TL and IgG-Tc antibodies were taken from blood samples. Restricting the dataset to include only those faecal and blood samples collected on the same day (94.22% of samples) did not qualitatively change the results.

#### (i) Strongyle faecal egg count

Parasite burdens in faecal samples collected each August were estimated using a modified McMaster technique. Briefly, 3 g of faecal sample was suspended in a saturated salt solution, and pipetted onto a McMaster slide. At 10× magnification, the number of strongyle eggs was counted and multiplied by 100 to estimate the FEC per gram of sample (complete details in [63]). Previous studies have shown a positive, linear association between FEC and the parasite burden of adult nematodes in individuals examined post-mortem, indicating FEC to be a good proxy of overall strongyle burdens in this population [39,40].

#### (ii) IgG-Tc antibodies

Blood samples were collected in lithium heparin tubes and kept in a cool box and 4°C fridge until processing, within 24 h of sampling. The vacutainer tubes were spun at 1008 × *g* for 10 min, after which the plasma layer was removed and replaced by the same quantity of 0.9% NaCl solution, and spun again at 1008 × *g* for 10 min. The buffy coat layer, comprising mainly white blood cells, was then transferred to a 1.5 ml Eppendorf tube and stored at −20°C.

The levels of IgG antibodies against antigens of the third larval stage of *T. circumcincta* (IgG-Tc antibodies) were measured using direct enzyme-linked immunosorbent assays (ELISAs) following established protocols [43]. In brief, diluted *T. circumcincta* L3 somatic antigen (capture antigen in the assays) was added to every well of a 4°C incubated 96-microwell plate. After plate washing using Tris-buffered saline-Tween (TBST), diluted Soay sheep plasma samples were added to each well followed by incubation for 1 h at 37°C. This was followed by another set of TBST washing after which diluted rabbit anti-sheep IgG detection antibody conjugated to horseradish peroxidase (HRP; AbD Serotec 5184–2104) was added to the IgG assay. Plates were subsequently incubated at 37°C for 1 h followed by another set of TBST washing. SureBlue TMB 1-Component microwell peroxidase substrate was then added to every well and plates were incubated in the dark for 5 min at 37°C followed by addition of 1 M hydrochloric acid to stop the reactions. Optical densities (ODs) were immediately estimated at 450 nm using a Thermo Scientific GO Spectrophotometer and recorded. Every plate also included negative controls (two wells containing only TBST with no sheep plasma sample) and positive controls (two wells with plasma from healthy, non-immunized domestic sheep) to minimize within-plate variation. The mean optical density ratio was used for all subsequent analyses and calculated according to

$$\mathrm{OD}=\;\frac{\mathrm{sample}\;\mathrm{OD}-\mathrm{blank}\;\mathrm{OD}}{\mathrm{positive}\;\mathrm{control}\;\mathrm{OD}-\mathrm{blank}\;\;\mathrm{OD}}.$$

To reduce confounding due to capture year and age with respect to plate-to-plate variation, every plate included samples from 2 years randomly paired with different ages on every plate. Plates were run in duplicate and duplicate sample ODs removed if the coefficient of variation (COV) was > 0.2 OD units. Correlation of ODs across duplicate plates was calculated and if found to be less than 0.8, both plates were rerun with all duplicate plates passing this threshold (complete details in [43]).

#### (iii) Relative leucocyte telomere length

Genomic DNA was extracted from the buffy coat layer of the blood samples using the Macherey-Nagel Nucleospin 96 Blood kit (cat. no. 740665). This was performed on 96-well plates and the samples were eluted to a final volume of 150 µl in elution buffer available from the kit. The relative leucocyte TL (amount of telomeric DNA sequence relative to amount of the reference gene, beta-2-microglobin) was measured using real-time qPCR based on methods validated previously in sheep and cattle blood samples [49,64]. On the same qPCR plate, telomere samples and reference gene samples were run in separate wells. Eight calibrator samples were also included on every plate to account for variation among plates. 2 non-template controls (NTCs) prepared with nuclease-free water were also included on each plate. A standard curve was estimated using serially diluted samples of the calibrator and all samples were run in triplicate.

Using the LinRegPCR software package (version 2016.0) [65], baseline fluorescence correction of the amplification curves was calculated. This software was also used to calculate well-specific reaction efficiencies and cycle quantification (Cq) values. Samples were excluded from further analysis based on a 5% threshold for the COV across triplicates and/or PCR efficiency values for a respective amplicon.

We calculated relative leucocyte telomere length (hereafter RLTL) for each sample (following [66]). Average reaction efficiencies for each plate and Cq for each sample were determined by LinRegPCR, and then RLTL was calculated as follows:

$$\mathrm{RLTL}=\;\frac{E_{TEL}^{\left(Cq_{TEL}[\mathrm{calibrator}]-Cq_{TEL}[\mathrm{sample}]\right)}}{E_{B2M}^{\left(Cq_{B2M}[\mathrm{calibrator}]-Cq_{B2M}[\mathrm{sample}]\right)}},$$

where $E_{TEL}$ and $E_{B2M}$ are the mean reaction efficiencies for the respective amplicon group across all samples on a given plate; $Cq_{TEL}[\mathrm{calibrator}]$ and $Cq_{B2M}[\mathrm{calibrator}]$ are the average Cqs for the relevant amplicon across all calibrator samples on the plate; and $Cq_{TEL}[\mathrm{sample}]$ and $Cq_{B2M}[\mathrm{sample}]$ are the average of the triplicate Cqs for the sample for each amplicon. We estimated the technical repeatability of RLTL to be 0.866 (see [48] for further details).

### (c) Data analysis

We investigated the associations between RLTL, parasite burden and antibody levels separately for lambs (approx. 4 months of age) and adults (≥ 3 years of age). This was because the physiological costs of infection and immunity could potentially differ between these life stages. We also reran the adult models using data from the entire adult population (adults aged ≥ 1 years old) to maximize sample size. Since each of the datasets (RLTL, FEC and IgG-Tc) had different proportions of missing data, the sample sizes for datasets combining these different variables also varied. The number of observations across both age groups and from each sex for the different datasets are detailed in electronic supplementary material, table S1.

Our analysis in lambs involved using univariate linear mixed-effect models (LMMs) to test costs of infection and immunity on RLTL. In adults, we used multivariate GLMMs to understand the association between RLTL and parasitism, and RLTL and helminth-specific antibody levels. Using the multivariate approach, we can not only decompose sources of variance (at the among-individual, among-year and within-individual levels) in each response variable, we can also estimate the covariance across variables at these different levels. This allowed us to directly estimate the covariance at the among- and within-individual level (as well as other levels fitted as random effects). This modelling approach to separate within- and among-individual processes is expected to give equivalent results to the more widely used within-subject centring approach to separate within- and among-individual processes [44,48,67,68]. Furthermore, it offers several statistical advantages over the within-subject centring approach, which include (a) accounting for error associated with the estimation of individual means, (b) estimating covariances across different response variables and making no assumptions about the causal direction of the effect, and (c) accounting for potential covariances by including additional response variables in the model and across different levels of random effects.

#### (i) Costs of parasitism and immunity in lambs

We used univariate LMMs to test if RLTL was predicted by FEC and IgG-Tc antibody levels in lambs (*n* = 831 observations for FEC; *n* = 1103 observations for IgG-Tc). We ran separate LMMs including strongyle FEC and antibody levels as predictors, respectively. To account for inter-annual variation in RLTL, we included year of measurement as a random effect. We also accounted for measurement error associated with RLTL qPCR assays by including qPCR plate and qPCR row as additional crossed random effects (following [48]). We tested for interactions between sex and FEC or sex and IgG-Tc in our models of RLTL. These models also included sex as a categorical predictor (according to the principle of marginality [69]). Since circulating IgG-Tc antibody levels may influence and be correlated with overall parasite burden [43], we also ran a single LMM which included both strongyle FEC and IgG-Tc as predictors to understand whether FEC and IgG-Tc were independently associated with RLTL (*n* = 828 observations). The LMMs were run using Bayesian statistical modelling implemented in Stan using ‘rstanarm’ v 2.21.1 [70]). Details of implementation of this analysis is described in the electronic supplementary material, Methods.

#### (ii) Parasitism costs and immunosenescence in adults

To understand how costs of parasitism and immunosenescence may influence leucocyte TL in adults, we fitted multivariate GLMMs of RLTL, FEC and IgG-Tc using a Bayesian framework, implemented in the R package ‘MCMCglmm’ v 2.29 [71]. Using multivariate models, we were able to estimate the variance of each of the response variables (RLTL, FEC and IgG-Tc) as well as the covariance between them at different grouping levels [44,48]. We were interested here in understanding the contributions of among-individual, among-year and within-individual processes underlying the associations of leucocyte TL with strongyle FEC and IgG-Tc antibody levels. We ran a single trivariate model with RLTL, strongyle FEC and IgG-Tc antibodies as the three response variables. With this, we were able to estimate the 3 × 3 variance–covariance matrix of RLTL, FEC and IgG-Tc for each of the random effects (individual and year) and the residual variance, and calculate the correlations where

$$\mathrm{correlation}=\frac{\mathrm{covariance}(\mathrm{Trait}\;1,\mathrm{Trait}\;2)\;}{\mathrm{SD}(\mathrm{Trait}\;1)*\mathrm{SD}(\mathrm{Trait}\;2)}.$$

For RLTL, the total variance in RLTL can be thought to be the sum of (a) differences among individuals in their mean RLTL (also called ‘among-individual’ variance), (b) annual differences in RLTL across the population, (c) assay-specific variance and (d) residual variance. The residual variance captures deviations from the mean RLTL of individuals across multiple measurements, or the ‘within-individual’ processes [44,48]. Similarly, for FEC and IgG-Tc, the total variance in these traits is a sum of multiple components, namely (a) among-individual variance, (b) annual variance, (c) assay-specific variance and (d) within-individual (i.e. residual) variance. The covariances between RLTL, FEC and IgG-Tc can also be estimated at the among-individual, among-year and within-individual levels.

For this analysis, we used RLTL, FEC and IgG-Tc antibody data from adults aged ≥3 years old, since a previous study found immunosenescence within this adult sub-population [44]. This analysis included all observations for which complete information regarding RLTL, FEC and IgG-Tc was known (*n* = 1348 observations from 572 individuals). The multivariate model included random effects for individual identity and year fitted for all three response variables (RLTL, FEC, IgG-Tc) using unstructured variance–covariance matrices:

$$\sigma_{\mathrm{individual}}^{2}=\left[\begin{array}{ccc} \sigma_{RLTL_{ID}}^{2} & \sigma_{RLTL_{ID},FEC_{ID}} & \sigma_{RLTL_{ID},IgG_{ID}}\\ \sigma_{RLTL_{ID},FEC_{ID}} & \sigma_{FEC_{ID}}^{2} & \sigma_{FEC_{ID},IgG_{ID}}\\ \sigma_{RLTL_{ID},IgG_{ID}} & \sigma_{FEC_{ID},IgG_{ID}} & \sigma_{IgG_{ID}}^{2} \end{array}\right],$$

$$\sigma_{\mathrm{year}}^{2}=\left[\begin{array}{ccc} \sigma_{RLTL_{Yr}}^{2} & \sigma_{RLTL_{Yr},FEC_{Yr}} & \sigma_{RLTL_{Yr},IgG_{Yr}}\\ \sigma_{RLTL_{Yr},FEC_{Yr}} & \sigma_{FEC_{Yr}}^{2} & \sigma_{FEC_{Yr},IgG_{Yr}}\\ \sigma_{RLTL_{Yr},IgG_{Yr}} & \sigma_{FEC_{Yr},IgG_{Yr}} & \sigma_{IgG_{Yr}}^{2} \end{array}\right]$$

$$\mathrm{and}\;\sigma_{\mathrm{residual}}^{2}=\left[\begin{array}{ccc} \sigma_{RLTL_{Res}}^{2} & \sigma_{RLTL_{Res},FEC_{Res}} & \sigma_{RLTL_{Res},IgG_{Res}}\\ \sigma_{RLTL_{Res},FEC_{Res}} & \sigma_{FEC_{Res}}^{2} & \sigma_{FEC_{Res},IgG_{Res}}\\ \sigma_{RLTL_{Res},IgG_{Res}} & \sigma_{FEC_{Res},IgG_{Res}} & \sigma_{IgG_{Res}}^{2} \end{array}\right].$$

qPCR plate and row within qPCR plate were also included as crossed random effects in the RLTL model. ELISA plate was included as a random effect in the IgG-Tc model. We obtained a posterior distribution for the variance and covariance between our response traits at the among-individual, among-year and residual levels. For RLTL, age was fitted as a fixed effect. For FEC, we included age (linear and quadratic terms) and sex as fixed effects. For IgG-Tc, age (linear term) and sex were fitted as fixed effects. RLTL and IgG-Tc were modelled using Gaussian distributions with strongyle FEC fitted using a negative binomial distribution. The model was implemented using MCMCglmm and run for 4.95 × 10^5^ iterations, with 9.5 × 10^4^ iterations as warm-up and a thinning interval of 400 resulting in a posterior stored sample size of 1000. Parameter expanded priors were used for the variance components with variances of 1, prior means of 0 and prior covariances of 1000. For the residual variances in all models, inverse-Wishart priors were used. Prior to inclusion in the model, RLTL, IgG-Tc and age were standardized to mean = 0 and standard deviation = 1. FEC was rounded to the nearest whole number and divided by 100. Autocorrelation was calculated to be < 0.1 for all parameters in the models. We report point estimates as the mode of the posterior distribution and the uncertainty as the 95% highest posterior density intervals (credible intervals; CIs). Terms were considered statistically significant based on their 95% CIs not spanning 0. Further details regarding the estimation of repeatability are available in Supplementary Methods. We also reran this model using data from all adult individuals (≥ 1 year of age) with the results presented in electronic supplementary material, table S10. Additionally, we reran this model excluding age as a fixed effect with these results presented in electronic supplementary material, table S13.

We also hypothesized that the costs of parasitism and immunity on RLTL in adults could be sex-specific. We tested this by running two univariate LMMs of RLTL which included effects of FEC or IgG-Tc, respectively, and their interaction with sex. We included the age of the adult as a fixed covariate, and random effects of individual, year, qPCR plate and qPCR row to account for repeated measures. These models were implemented in the same way as the univariate analyses in lambs.

#### (iii) Relationship between RLTL, FEC, IgG-Tc and survival

To test whether RLTL was a potential mediator of associations between infection and annual overwinter survival probability and/or a marker of overall immune status, we ran a multivariate GLMM of RLTL, strongyle FEC, IgG-Tc antibodies and annual survival implemented in the R package ‘MCMCglmm’ v 2.29 [71]. To aid model convergence and maximize statistical power, we used data from the entire population. This included lambs (age 0) and all adults (≥ 1 year of age) for whom complete information regarding RLTL, FEC, IgG-Tc and survival was known (*n* = 2879 observations from 1366 individuals). In this analysis using data from the entire population, 44.58% of individuals had been sampled more than once. The average number of samples per individual was 2.11. In total, there were 757, 240, 149 and 220 individuals sampled once, twice, thrice and ≥4 times, respectively. We controlled for age in these models by including age-class (2-level factor distinguishing between lambs or adults) and age in years (as a covariate; further details in electronic supplementary material, Methods). With this multivariate model, we were able to estimate the covariance between RLTL, FEC, IgG-Tc and annual survival at different hierarchical levels. Subsequently, using a multiple regression approach, we obtained the partial regression coefficients from the multiple regression of the different predictors (RLTL, FEC and IgG-Tc) on survival [44,59]. These regression estimates represented the effect of each predictor on survival, after accounting for the covariance between the predictors. Further details of model structure and implementation are available in the electronic supplementary material, Methods.

## 3. Results

### (a) Costs of parasitism and immunity in lambs

**Figure 1.**
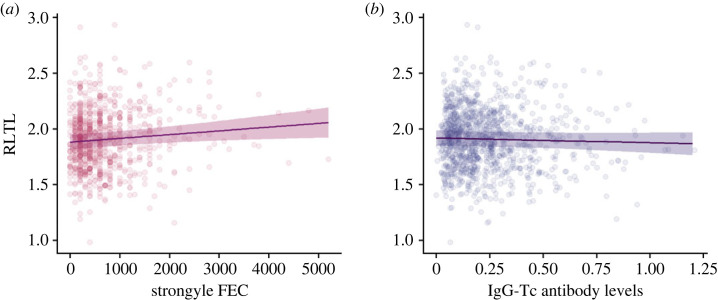
Relationship between relative leucocyte telomere length (RLTL) and (*a*) strongyle FEC (*n* = 831 observations) and (*b*) IgG-Tc antibody levels (*n* = 1103 observations) in Soay sheep lambs. Points represent raw data and the median line (in bold) and 95% credible intervals are from the posterior distribution. Full model estimates in electronic supplementary material, tables S2 and S3.

We found no support for leucocyte TL reflecting a cost of parasitism in lambs, which would predict a negative association between RLTL and FEC (n = 831 observations). Instead, we found support for a positive association between RLTL and strongyle FEC (estimate: 3.36 × 10^−5^, 95% CI: 6.92 × 10^−6^–6.05 × 10^−5^; figure 1*a*). There was no evidence for a relationship between RLTL and IgG-Tc (*n* = 1103 observations), and thus no support for leucocyte TL reflecting a cost of immune defence in lambs (estimate: −0.043 and 95% CI: −0.126–0.040; figure 1*b*). Full model parameter estimates and their 95% credible intervals (CIs) are shown in electronic supplementary material, tables S2 and S3.

There was also no support for independent costs of parasitism or immune defence on RLTL in a model containing both terms. Instead, a positive correlation between RLTL and FEC was observed even after accounting for variation in IgG-Tc levels (estimate: 3.31 × 10^−5^; 95% CI: 5.71 × 10^−6^–6.03 × 10^−5^; electronic supplementary material, table S4). We found no support for any sex-specific differences in the relationship between RLTL and parasite burdens, and RLTL and IgG-Tc antibody response. The 95% CIs spanned 0 for all interaction terms in these models (electronic supplementary material, table S5 and S6).

### (b) Parasitism costs and immunosenescence in adults

**Figure 2.**
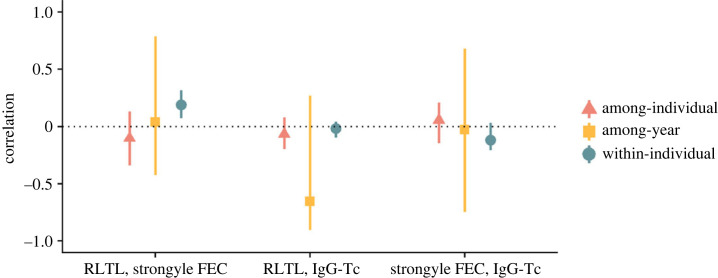
Correlations between relative leucocyte telomere length (RLTL), strongyle FEC and IgG-Tc (*n* = 1348 observations from 572 individuals) at different hierarchical levels in Soay sheep adults (aged ≥ 3 years). Correlations were estimated as the mode of the posterior distribution with 95% CIs from multivariate Bayesian mixed-effects models. Estimates for among-individual, among-year and within-individual (residual) correlations displayed (full model estimates in electronic supplementary material, table S7).

In adult sheep (≥ 3 years old; *n* = 1348 observations from 572 individuals), our multivariate mixed models confirmed that there were repeatable, among-individual differences in RLTL, strongyle FEC and IgG-Tc (repeatability of RLTL: 0.254, 95% CI: 0.184–0.316; repeatability of FEC: 0.375, 95% CI: 0.258–0.517; repeatability of IgG-Tc: 0.595, 95% CI: 0.537–0.659; electronic supplementary material, figure S1). However, there was no evidence for among-individual or among-year covariance among RLTL, FEC and IgG-Tc (electronic supplementary material, table S7). Therefore, there was no association between average adult RLTL and average adult FEC or IgG-Tc levels in this population. We did find support for a small positive within-individual correlation between RLTL and strongyle FEC, independent of IgG-Tc antibody levels (0.190; 95% CI: 0.073–0.316; figure 2; electronic supplementary material, table S7). This suggests individuals with high parasite burdens also had longer RLTL within years. Thus, we found no support for costs of infection on RLTL in adults. There was no evidence of any within-individual covariance between RLTL and IgG-Tc (figure 2; electronic supplementary material, table S7). We therefore found no support for variation in RLTL underlying immunosenescence patterns in adults nor did we find costs of immunity on RLTL. There was also no evidence of sex-specific costs of parasitism or immunity on RLTL in adults (electronic supplementary material, table S8 and S9). The main findings were unchanged when we reran this model including all adult individuals (≥ 1 years of age; n = 2097 observations from 846 individuals), finding a positive within-individual correlation between RLTL and FEC (0.143; 95% CI: 0.048–0.231; electronic supplementary material, table S10).

### (c) Relationship between RLTL, FEC, IgG-Tc and survival

**Figure 3.**
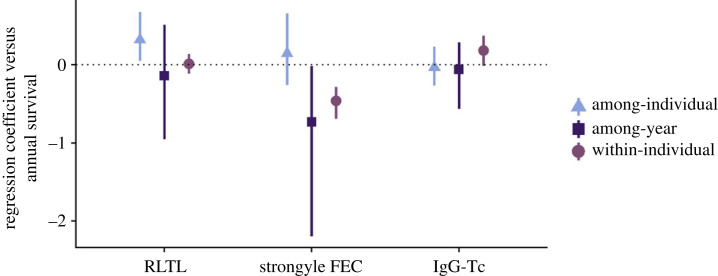
The partial regression coefficients obtained from a multivariate model of RLTL, strongyle FEC, IgG-Tc and overwinter survival using data from the entire Soay sheep population (*n* = 2879 observations from 1366 individuals). The effect of different traits on survival is shown, while accounting for the covariance between these traits at different hierarchical levels. Mode of the posterior distribution with 95% credible intervals depicted (full model estimates in electronic supplementary material, tables S11 and S12).

Using data from the entire population (*n* = 2879 observations from 1366 individuals), we found RLTL, FEC and IgG-Tc to each independently predict overwinter survival. RLTL and survival were positively associated at the among-individual level (0.318; 95% CI: 0.051–0.676; figure 3; electronic supplementary material, table S11), FEC was negatively associated with survival at both the among-year and within-individual levels (among-year: −0.732; 95% CI: −2.195 to −0.0164; within-individual: −0.461; 95% CI: −0.695 to −0.284; figure 3; electronic supplementary material, table S11). IgG-Tc was positively associated with survival at the within-individual level although the 95% CIs very narrowly crossed 0 (0.181; 95% CI: −0.014–0.369; figure 3; electronic supplementary material, table S11). These associations were observed after accounting for the covariance between these predictors suggesting independent effects of these factors on subsequent survival. This suggests that across lambs and adults, associations between infection or immunity and survival are not mediated via leucocyte RTL nor does leucocyte RTL act as a marker of helminth-specific antibody immune status.

## 4. Discussion

We found no correlative support for the hypothesis that costs of infection manifest as decreased RLTL in Soay sheep lambs or adults In fact, we found the opposite with strongyle parasite burdens being weakly positively associated with RLTL in both age groups. The positive association was present even after accounting for variation in IgG-Tc antibody levels, suggesting that the association between parasite burdens and RLTL in this population acts independent of the IgG-Tc antibody-mediated immune response. This suggests mechanisms unrelated to antibody-mediated immunity to worms underpin the association between RLTL and FEC. We found no correlative evidence for immunity-associated costs on RLTL in lambs, finding no association between RLTL and the IgG-Tc antibody immune response. There was also no correlation between RLTL and IgG-Tc in adults, indicating no support for RLTL mediating immunosenescence. Using data from the entire population, we employed a multiple regression approach adopted within a multivariate model framework to evaluate whether leucocyte TL mediated the association between parasite burdens, immune-related traits and overwinter survival. Confirming previous work in this system, we found a positive covariance between RLTL and overwinter survival (at the among-individual level) and a negative covariance between FEC and overwinter survival (at the among-year and within-individual levels), even after accounting for covariances with IgG-Tc [44,48]. There was no evidence that variation in leucocyte TL might be a physiological mechanism underpinning the covariance between parasitism and survival in this population. There was also no support for leucocyte TL reflecting an individual's overall antibody immune status since the positive covariances of survival with leucocyte TL and IgG-Tc were independently present, even after accounting for covariances with strongyle FEC.

Previous studies in natural populations have found varied support for the predicted negative relationship between parasite burden and TL. In humans, studies have found individuals with chronic viral infections to have shortened TLs [26,27]. There was no cost of haemosporidian infection on TL in some wild bird species [22,24]. Similarly, a study in wild fish found no association between parasite burdens and TL [23]. However, in other wild birds, individuals infected with haemosporidian parasites had faster TL shortening and/or shorter TLs, compared to uninfected individuals [5,6,21]. A cost of bacterial infection on TL was also identified in wild house mice (*Mus musculus*) [10]. A previous study in wild badgers (*Meles meles*) found individuals with positive TB infection status had shorter TLs than individuals who tested negative. In addition, individuals in advanced stages of TB infection were found to have shorter TLs than individuals who tested positive for TB [7]. Our study revealed a positive association between leucocyte TL and parasite burdens in wild sheep. Although we acknowledge that identifying infection costs in a non-experimental study is somewhat limited in scope, we failed to detect costs of parasitism on TL within the natural continuum of parasite burdens in our population. This highlights the very complex relationship between parasitism and TL in the wild with a wide variety of outcomes observed across diverse taxa. This suggests TL to be a highly context-specific marker of infection costs in the wild.

The relationship between TL and infection may be influenced by a suite of different factors. For instance, host-specific processes such as variation in innate and adaptive immune responses across different taxa as well as variation in TL dynamics and telomerase expression throughout the animal kingdom could result in wide-ranging associations between parasitism and TL [72–74]. Additionally, associations may depend on the type of parasite (e.g. microparasites or macroparasites; endo- or ecto-parasites), type of infection and the immune responses parasites induce in their hosts (acute versus chronic infections; Th1 versus Th2 immune responses) and also the degree of co-infection with multiple parasites [75–77]. In our system, chronic infection with strongyle nematodes is not as strongly associated with leucocyte TL as in some avian studies linking malaria infection and erythrocyte TL. Since malaria parasites directly infect and influence the replication of erythrocytes, this is unsurprising [5]. Instead, the positive association we identified between strongyle parasite burdens and RLTL could potentially be driven by other (measured or unmeasured) variables that may be correlated with both high parasite burdens and long TLs. For instance, parasite exposure in lambs that have yet to acquire immune protection may be positively linked to habitat quality [40]. Sheep inhabiting high-quality forage areas could be exposed to high levels of infective strongyle larvae due to high host population density in these habitats [40,78,79]. These individuals may also be able to maintain long leucocyte TLs due to the abundant resources in these high-quality habitats, resulting in a positive association between parasite burdens and leucocyte TL. Additionally, in adults, high worm burdens may potentially lead to an attenuated overall immune response (that is not reflected in IgG levels) and decreased leucocyte cell divisions, contributing to longer leucocyte TLs [80]. Lamb mortality during the neonatal period can vary between 10 and 40% in this population, with almost 85% mortality occurring within the first month of birth [60,81,82]. However, a previous study in females in this population found no evidence of selective disappearance of lambs with short TL between birth and the following summer [49]. There could be potential effects of ancient domestication on leucocyte TLs and parasite burdens in this population [42,83]. Further investigation of just how generalizable the hypothesis that infection negatively impacts TL is needed to understand the role of host–parasite system-specific mechanisms on TL. We will also benefit from understanding the ecological relevance of TL and how variation in the resistance to infection and maintenance of TL has evolved together with studies conducted in natural settings.

Studies investigating the relationship between immunity and TL in natural populations report contrasting findings. In healthy human adults, individuals with shorter TL had decreased resistance towards experimentally-induced acute respiratory infection [84]. In purple-crowned fairy wrens (*Malurus coronatus*), TL was found to be positively associated with a specific component of the innate immune response at the among-individual level but unrelated to other innate immune indices [8]. In European wild badgers, there was no association between leucocyte TL and IFN-ɣ (involved in both the innate and adaptive immune response) [38]. We similarly found no association between leucocyte TL and IgG-Tc in both lambs and adults in our population and this suggests that worm-specific IgG-mediated antibody immune response and leucocyte TL dynamics are unrelated in this system. It is possible that four-month old lambs produce a relatively weak overall immune response either due to an immature immune system as a result of limited exposure to parasites, active immunosuppression or investment in other nutrient-demanding activities such as growth and thermoregulation [17,43,85,86]. The effect of variation in early-life immune response and investment in leucocyte TL may therefore be muted. By adulthood, antibody immune responses to chronic helminth parasite infections are fully established and largely considered to operate via tolerance mechanisms. The links between tolerance-based immunity and leucocyte TL are unknown [78]. Furthermore, all leucocyte cell types contribute to the leucocyte TL measure in our study (and others like it). The telomere dynamics of different immune cell types are known to differ, in part due to variation in proliferation rates and telomerase expression [87–89]. This may cloud any signal associated with the TL dynamics of the B lymphocytes underlying the IgG-Tc-mediated antibody response. That said, a previous study in the Soay sheep using a smaller dataset found no correlation between leucocyte RTL and ratios of different immune cell subtypes [90]. Although a study in humans suggests TL from different hematopoietic cells to be correlated, measuring TL in specific cell subtypes may be needed to accurately determine the association between the antibody-mediated immune response and TL [91].

Our multivariate modelling approach revealed that the positive relationship between RLTL and FEC occurred at the within-individual level. This means that short-term, annual variation in RLTL and strongyle FEC within individuals are correlated, rather than consistent differences among individuals due to, for instance, genetics and early-life environment. This suggests recent physiological variation (such as year-to-year variation in parasite burdens within individuals or recent investment in reproduction) or short-term environment-related effects affecting resource availability contribute to the positive covariance between RLTL and worm burdens. Furthermore, since the positive covariance between RLTL and FEC manifested at the within-individual level alone, there was no support for any causal association between RLTL and FEC in this population [48,59]. This is because any causal association would be expected to manifest at all hierarchal levels (among-individual, among-year and within-individual). If causally related, individuals with low parasite burdens should have short leucocyte TL both on average across their lifetime and within a given year compared to their lifetime mean TL. Although previous studies suggest TL captures costs of infection and trade-offs between different life-history traits, we found RLTL to neither be involved in mediating any trade-offs between infection and survival nor representing the antibody-mediated immune response of individuals in this population since each of these predictors (RLTL, strongyle FEC and IgG-Tc) independently predicted survival. Employing this multivariate modelling approach in other longitudinal wild animal datasets could improve our understanding of the links between TL, infection, immunosenescence and subsequent survival.

In conclusion, we found no correlative evidence for potential costs of parasitism on leucocyte TL in the Soay sheep. Although studies in eco-immunology have identified costs of parasitism and immune defence manifesting on life-history traits like future survival and reproduction, it is evident from our study that such fitness costs, although observed in this population, are not reflected by variation in leucocyte TL in this correlational study [17,58,92]. This study provides further evidence supporting TL to be weakly linked to parasite burdens in the wild and the association between leucocyte TL, parasitism and immunity to be highly context dependent. More longitudinal studies evaluating these relationships in wild populations across different taxa are needed to understand just how useful of a biomarker TL is under natural conditions which would further our understanding of whether associations between TL and different life-history traits are generalizable across the animal kingdom [11].

## Data Availability

The data and R code used in this study are available at https://doi.org/10.5281/zenodo.10839266 [93]. Supplementary material is available online [94].
